# A New Approach Using Manganese-Enhanced MRI to Diagnose Acute Mesenteric Ischemia in a Rabbit Model: Initial Experience

**DOI:** 10.1155/2015/579639

**Published:** 2015-11-26

**Authors:** Da-wei Zhao, Cheng Cheng, Lian-qin Kuang, Yu-long Zhang, Hai-yun Cheng, Jia-yan Min, Yi Wang

**Affiliations:** Department of Radiology, Institute of Surgery Research, Daping Hospital, Third Military Medical University, Chongqing 400042, China

## Abstract

*Purpose*. Manganese-enhanced MRI (MEMRI) has been applied to a wide range of biological and disease research. The purpose of the study was to use MEMRI to diagnose the acute mesenteric ischemia (AMI). *Methods*. The institutional experimental animal ethics committee approved this study. To optimize the dose of Mn^2+^ infusion, a dose-dependent curve was obtained using Mn^2+^-enhanced *T*_1_ map MRI by an intravenous infusion 2.5–20 nmol/g body weight (BW) of 50 nmol/L MnCl_2_. The eighteen animals were divided into control, sham-operated, and AMI groups. AMI models were performed by ligating the superior mesenteric artery (SMA). *T*_1_ values were measured on *T*_1_ maps in regions of the small intestinal wall and relaxation rate (Δ*R*_1_) was calculated. *Results*. A nonlinear relationship between infused MnCl_2_ solution dose and increase in small intestinal wall Δ*R*_1_ was observed. Control animal exhibited significant Mn^2+^ clearance over time at the dose of 15 nmol/g BW. In the AMI model, Δ*R*_1_ values (0.95 ± 0.13) in the small intestinal wall were significantly lower than in control group (2.05 ± 0.19) after Mn^2+^ infusion (*P* < 0.01). *Conclusion*. The data suggest that MEMRI shows potential as a diagnostic technique that is directly sensitive to the poor or absent perfusion in AMI.

## 1. Introduction

Acute mesenteric ischemia (AMI) is thought to be a life-threatening abdominal emergency characterized by the sudden occlusion of mesenteric arteries followed by impairment of intestinal blood flow. Currently, AMI accounts for approximately 0.1% of all hospital admissions, and it constitutes 1% to 2% of all gastrointestinal diseases [1]. The incidence has recently increased with the increasing age of the population [2].

The mortality rate of AMI is approximately 71%, and it has remained at this high level for decades [3]. The reasons for this are illustrated as follows. On one hand, early symptoms are nonspecific in differential diagnosis of abdominal pain; on the other hand, there is an unacceptable time delay before treatment even when a diagnosis of AMI is considered. Even a warm ischemia time of 6 hours leads to morphological alterations due to disintegration of the intestinal mucosal barriers, following the bacterial translocation and gangrene of the intestinal wall [2]. This eventually results in severe peritonitis, ileus, sepsis, and multiorgan failure. A 24-hour delay decreases survival rates by 20% [1]. Therefore, early diagnosis and development of new diagnostic strategy, before the development of intestinal infarction and peritonitis, are essential for intestinal viability and patient survival.

Currently, the clinical diagnosis for AMI is based on the vascular imaging to display the occlusive site of vessels through computer tomography angiography (CTA) and magnetic resonance angiography (MRA). Still, multidetector computer tomography (MDCT) has been currently the first-line and standard diagnostic method [4, 5]. MDCT demonstrates not only vascular structures but also intestinal wall changes and free abdominal air that may produce by hollow organ perforation [6]. This is substantial benefit for differential diagnosis between mesenteric ischemia and the other acute abdomen. The dynamic MRA yielded sensitivity and specificity of 95% and 100%, respectively [7]. In the study using an* in vivo* rat mesenteric ischemia model, 7-T MRI allows for the identification of pathological findings of ischemic colitis and histopathological correlation [8].

The high *T*_1_ relaxivity of Mn^2+^ and the ability of Mn^2+^ to enter cells through Ca^2+^ channels have led to the development of manganese-enhanced MRI (MEMRI) for a wide range of biological study, including brain anatomy and function [9–12], myocardial infarction [13, 14], and brain ischemia [15]. Therefore, in the present study, a strategy using MEMRI to diagnose AMI can be based on the fact that Mn^2+^ cannot arrive at suffered small intestine so that the relaxivity of small intestine will not be changed due to the reduction in contrast agents delivery caused by SMA in the AMI. Conversely, in the normal small intestinal wall without occlusion of SMA, due to the accumulation of Mn^2+^, the small intestine will have a shorter relaxivity, which in turn will increase signal intensity on *T*_1_-weighted image. We propose that MEMRI provide the direct image with the ability to distinguish between normal and ischemic small intestines for AMI.

## 2. Materials and Methods

### 2.1. Animal Subject and Preparation

The study was approved by the experimental animals ethics committee at Daping Hospital of Third Military Medical University. MEMRI experiments were performed in adult New Zealand white rabbits (mean ± SEM weight: 3.17 ± 0.12 kg), which were provided by the Animal Center of Daping Hospital. Rabbits were fasted for 12 hrs before experiment but were allowed free access to water. The animals were initially anesthetized with an intravenous injection of 3% pentobarbital sodium at the dose of 30 mg/kg. The anesthetized animals were maintained in side position on the warming pad throughout the MRI session. A custom-made abdominal bandage was used to provide abdominal pressure to prevent both abdominal breathing and intestinal peristalsis in an effort to minimize artifacts due to motion. During the whole procedure, animals maintained a relatively constant physiological level, body temperature (38.6 ± 0.71°C), heart rate (205.5 ± 12.90 beats/min), and breath frequency (35.0 ± 3.67 times/min).

A dorsal ear vein line for Mn^2+^ infusion was introduced after anesthesia. A 50 mM MnCl_2_ solution was obtained by dissolving MnCl_2_ crystals in saline (pH adjusted to 7.4). To acquire dose-dependent curve, MnCl_2_ was administered into control rabbits at the various doses ranging from 2.5 to 20 nmol/g total body weight (BW). For the temporal washout curve, a single dose of 20 nmol/g BW was infused into the control rabbits. All infusions were completed at a constant rate of 0.8 mL/min with the aid of a syringe pump (Baoding Lead Fluid Technology, China).

### 2.2. Acute Mesenteric Ischemia Model

For the AMI model study, the rabbits were randomly placed into the three following groups, with each group containing six rabbits: in control group, animals underwent no surgical procedures; in sham-operated group, animals underwent sham laparotomy with exposure of the superior mesenteric artery (SMA) after anesthesia; and, in AMI group, laparotomy was performed and the SMA were ligated. The AMI model was produced as literature previously described [16]. After anesthesia, the abdominal regions of the rabbits were sheared and sterilized with 10% povidone iodine and draped with sterile towels. Laparotomy was performed through a midline incision. After the small bowel and cecum were identified, the root of SMA was carefully exposed and ligated with 0 silk sutures. The abdominal contents were replaced, and then the peritoneum and abdominal wall were sutured following standard procedures. After surgery, MRI was immediately carried out according to the above-mentioned position.

For MnCl_2_ infusion, all three groups of rabbits were inserted into dorsal ear vein and infused with 15 nmol/g BW at a constant rate of 0.8 mL/min.

### 2.3. MRI

The* in vivo T*_1_ map MRI studies were carried out using anesthetized animals that had been placed in the magnet bore in the lateral position. In order to minimize artifacts due to motion, a custom-made abdominal bandage was used to achieve attenuating small bowel motion caused by both abdominal breathing and intestinal peristalsis.

All MR imaging was acquired at a 1.5 T MRI scanner (MAGNETOM Aera, Siemens AG, Germany) with a 70 cm bore magnet equipped with XI gradients (33 mT/m @ 125 T/m/s) and XQ gradients (45 mT/m @ 200 T/m/s). For signal excitation and reception, an abdominal phased array coil (20 mm, 63 MHz, Siemens AG, Germany) was used. A scout image was initially obtained in order to verify the proper position. Both pre-Mn^2+^ and post-Mn^2+^ *T*_1_ maps were acquired using *T*_1_ map sequence. The imaging parameters were as follows: TR/TE = 15/1.59 ms, slices per slab = 52, slice thickness = 3.0 mm, flip angle = 5° and 26°, matrix = 256 × 256, and FOV = 231 mm × 231 mm. MnCl_2_ was injected using a 22-gauge syringe through vein at a constant rate of 0.8 mL/min. After contrast agent injection, *T*_1_ map images were obtained approximately 5 min after completion of the infusion to allow Mn^2+^ blood pool clearance. The total imaging time per *T*_1_ map was 4 min and 50 s. *T*_1_ values were acquired before and after Mn^2+^ infusion to calculate the change of *T*_1_ relaxation rate due to MnCl_2_ infusion.

Both pre-Mn^2+^ and post-Mn^2+^ *T*_1_-weighted images were acquired using FLASH *T*_1_ dual echo sequence. The imaging parameters were as follows: TR = 250 ms, TE_1_ = 2.37 ms, TE_2_ = 4.87 ms, slices per slab = 52, slice thickness = 3.0 mm, number of averages = 1, matrix = 256 × 256, and FOV = 231 mm × 231 mm.

*T*_1_ map MRI datasets were processed offline for image reconstructions using workstation. ROI before and after Mn^2+^ infusion were defined in MRI anatomical and enhanced regions of small intestinal wall, respectively. For evaluation, *T*_1_ values of each rabbit were measured in small intestinal wall using defined ROI on the two-dimensional *T*_1_ maps to calculate average *T*_1_ values. The change of *T*_1_ relaxation rate (Δ*R*_1_) was calculated as post-Mn^2+^ infusion *R*_1_ − pre-Mn^2+^ infusion *R*_1_, where the relaxation rate (*R*_1_) is defined as 1/*T*_1_.

### 2.4. Statistical Analysis

All data are presented as mean ± SEM. Δ*R*_1_ values between the three groups in AMI study were compared by two-way ANOVA and Bonferroni post hoc test. All statistical analyses were performed using GraphPad Prism 5 (GraphPad Software Inc., San Diego, CA, USA). *P* value less than 0.05 was considered statistically significant.

## 3. Results

**Figure 1:**
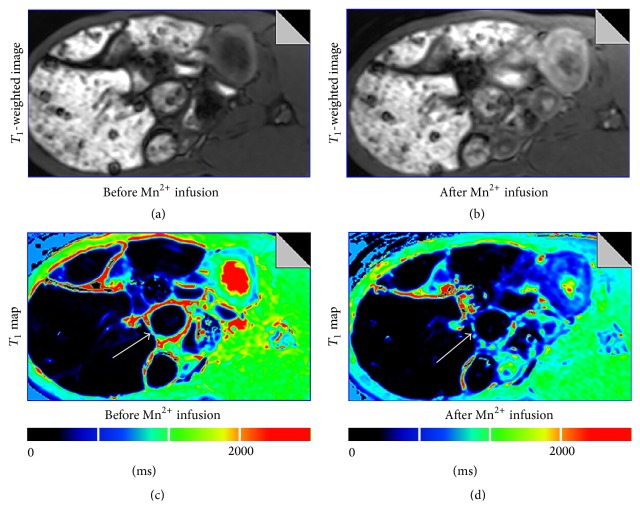
Example of small intestinal wall images showed the changes of  *T*_1_ signal in the small intestinal wall for a control rabbit in *T*_1_-weighted images and *T*_1_ maps. (a) *T*_1_-weighted image before Mn^2+^ infusion. (b) *T*_1_-weighted image after Mn^2+^ infusion. (c) *T*_1_ map before Mn^2+^ infusion. (d) *T*_1_ map after Mn^2+^ infusion. The white arrow indicated signal change of the small intestinal wall before and after Mn^2+^ infusion.

Sample small intestinal MR images for a control rabbit are shown in Figure 1. Figures 1(a) and 1(b) show *T*_1_-weighted signal intensity enhancements before and after Mn^2+^ infusion, respectively. Figures 1(c) and 1(d) show the corresponding *T*_1_ maps before and after Mn^2+^ infusion, respectively. At this Mn^2+^ dose of 15 nmol/g BW, there is significant enhancement of the signal intensity in the small intestinal wall after Mn^2+^ infusion.

**Figure 2:**
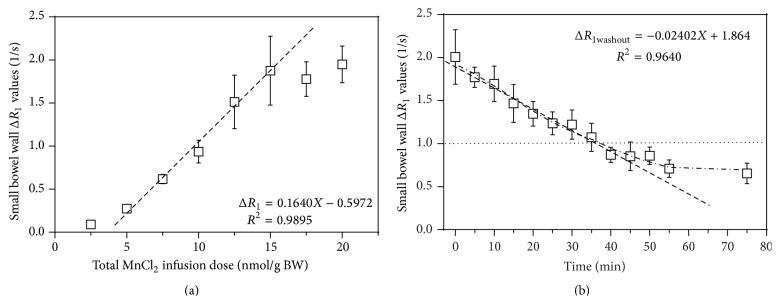
Effect of altering the dose of infused Mn^2+^ on small intestinal wall relaxivity. (a) The dose-dependent curve for different dose of MnCl_2_ administration. *x*-axis showed the total dose of infused Mn^2+^ normalized to rabbit BW. *y*-axis showed the change of relaxation rate, Δ*R*_1_. As a function of dose, a linear dose-uptake region was noted along with a plateau region above 15 nmol/g BW. The linear fit was shown with total Mn^2+^ infusion dose, *X*, in nmol/g BW. Data are presented as the mean ± SEM of *n* = 3. (b) Temporal Mn^2+^ washout curve. *x*-axis showed the experimental time course after Mn^2+^ infusion. *y*-axis showed the difference in the change of relaxation rate, Δ*R*_1_.

In present study, *T*_1_ map was performed to determine the relationship between Mn^2+^ infusion dose and tissue Δ*R*_1_ for small intestinal wall. The effect of altering the infusion dose on small intestinal wall Δ*R*_1_ is shown in Figure 2(a). This dose-dependent curve can be used to optimize minimal Mn^2+^ infusion doses while still achieving adequate signal enhancement. Δ*R*_1_ values ranged from 0.09/s at dose of 2.5 nmol/g BW to an average of 1.87/s for dose above 15 nmol/g BW. At Mn^2+^ infusion dose from 5 nmol/g BW to 15 nmol/g BW, the dose of Mn^2+^ infusion, *X* (nmol/g BW) on relaxivity, Δ*R*_1_ (1/s), yields a linear relationship of Δ*R*_1_ = 0.1640*X* − 0.5972 (*r* ^2^ = 0.9895). Above 15 nmol/g BW, the relaxivity enhancement reaches a plateau (Δ*R*_1_ = 1.87 ± 0.15/s). Within this physiological steady plane, any further rise in Mn^2+^ administered dose does not improve Δ*R*_1_.

Mn^2+^ temporal washout phenomenon was also studied in control rabbits infused with a single MnCl_2_ dose of 15 nmol/g BW. The data presented in Figure 2(b) show the temporal relationship between Δ*R*_1_ and the washout time. The washout period was examined from 0 to 75 min. The small intestinal wall Δ*R*_1_ signal has attenuated by 50% in the first 35 min after infusion. During the first 45 min after infusion, the washout data for this infusion dose can be fitted in a linear relationship with small intestinal wall Δ*R*_1 washout_ = −0.02402*X* + 1.864 (*r* ^2^ = 0.9640). This curve provides a better imaging time frame for the small intestinal disease model in this study.

**Figure 3:**
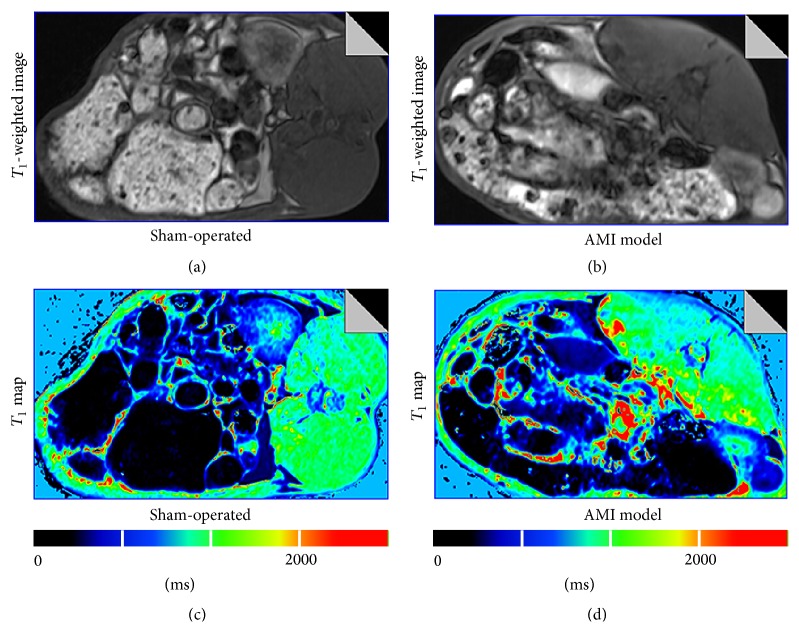
Example of post-Mn^2+^ infusion *T*_1_-weighted image and *T*_1_ map for a sham-operated rabbit (a and c) and AMI model (b and d). The small intestinal wall of AMI model showed a longer *T*_1_ relaxation time than that of sham-operated animal. Significant decreasing of *T*_1_ values can be seen in the small intestinal wall of sham-operated group compared with AMI groups.

The final aim of this study was to explore variety of relaxivity due to acute mesenteric ischemia. All three groups of rabbits, namely, control group, sham-operated group, and AMI model, were infused with a single MnCl_2_ dose of 15 nmol/g BW. Each group contained six animals. *T*_1_ mapping was immediately performed after initial Mn^2+^ infusion.  Figure 3 showed changes of signal on *T*_1_-weighted image and *T*_1_ maps for sham-operated group (Figures 3(a) and 3(c)) and AMI model (Figures 3(b) and 3(d)) after Mn^2+^ infusion. By comparing AMI model to the sham-operated group, there was a significant difference of signal enhancement. In the AMI model, there were higher *T*_1_ values on *T*_1_ map due to reduced uptake of Mn^2+^. On the contrary, in the sham-operated rabbits, there were lower *T*_1_ values on *T*_1_ map, accompanied by higher signal enhancement on *T*_1_-weighted image due to uptake of Mn^2+^.

**Figure 4:**
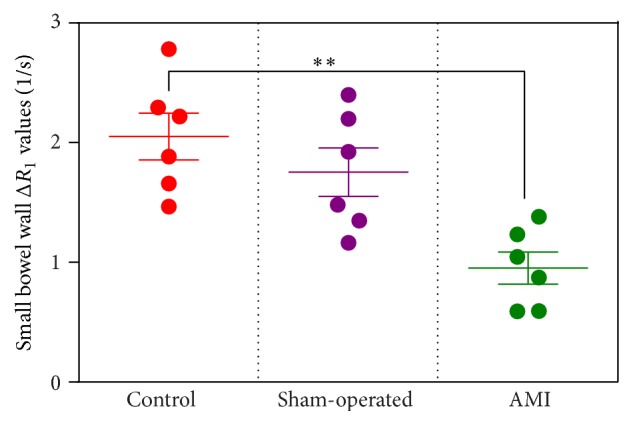
Effect of AMI on the uptake of Mn^2+^. Data are presented as the mean Δ*R*_1_ ± SEM of *n* = 6. ^*∗∗*^ *P* < 0.01 versus control group.

The results of this study were shown in Figure 4 and Table 1. There was no statistically significant difference in Δ*R*_1_ values between the control group (2.05 ± 0.19) and sham-operated group (1.75 ± 0.20), suggesting that the surgical operation of opening the abdomen does not affect Mn^2+^ uptake. Comparing AMI group with the control group, however, revealed that there was a significant difference in Δ*R*_1_ values after Mn^2+^ infusion. Δ*R*_1_ values of AMI groups (0.95 ± 0.13) were distinctly lower than those of control group (2.05 ± 0.19). These data demonstrate the sensitivity of this technique to determine altered Mn^2+^ uptake at the AMI model. Detecting changes of relaxivity in AMI injury may allow us to timely identify acute AMI intestinal wall in preclinical models for future treatment or prevention.

## 4. Discussion

AMI are vascular emergencies that require an immediate diagnosis and medical and surgical intervention. The preliminary data from this study reveal that normal and ischemic intestines can be identified using MEMRI technique in the rabbit AMI model. Additionally, the dosage of Mn^2+^ infusion for optimal signal enhancement in the small intestinal wall is first established via showing the dynamic range of relaxivity changes over a range of Mn^2+^ infusion doses. The dose-dependent curve reveals the nonlinear relationship between total Mn^2+^ infusion dose and Δ*R*_1_ of the small intestinal wall (Figure 2(a)). At a dose of above 15 nmol/g BW, a plateau of Δ*R*_1_ occurs, where Δ*R*_1_ does not increase after an increase in dose of Mn^2+^ infusion. This phenomenon is similar to the performance of Mn^2+^ in the heart reported by previous literature [14]. However, the dosages of Mn^2+^ reaching the plateau in the small intestinal wall are far less than those in the heart. Furthermore, the signal washout curve shows that the small intestinal wall Δ*R*_1_ signal has attenuated by 50% after 35 min (Figure 2(b)).

The known cytotoxic effects of high doses of Mn^2+^ initially discouraged its use as an MRI contrast agent [17]. Thus, minimizing the administered dose of Mn^2+^ is critical for MEMRI into clinical application. Here we have shown that a dose of 15 nmol/g BW, which is far less than 197 nmol/g BW described in other reports [14], can be selected to produce significant relaxivity changes in the small intestinal wall. Additionally, any further increase in the dose of Mn^2+^ above 15 nmol/g BW does not result in a further decrease in *T*_1_ values. Overall, these data suggest that Mn^2+^ is safe to use as a contrast agent in intestinal MEMRI.

Mn^2+^ can influence intestinal motility via affecting Ca^2+^ influx of the smooth muscles of the intestinal mucosa. Mn^2+^ affects Ca^2+^ influx in a concentration-dependent manner. Mn^2+^ inhibited smooth muscle contraction by blocking Ca^2+^ influx [18]; the inhibition could be overcome by high extracellular Ca^2+^ concentrations at millimolar concentrations [19]. Mn^2+^ can cause a transient contractile response through the release of acetylcholine [20] or directly induce contractions via voltage-operated L-type Ca^2+^ channels at millimolar concentrations [21–23]. In present study, the Mn^2+^ concentrations are at nanomolar range and are far lower than the concentrations described above.

The AMI study which used the optimal dose shows statistically significant differences to discern normal and ischemic small intestines using MEMRI between sham-operated and AMI groups. MEMRI had previously been shown to provide functional and pathological information in myocardial tissue [13, 14, 24] and brain [12, 25]. AMI caused by the occlusion of SMA show a no-flow status in the suffering small intestine. These changes can potentially be observed and monitored using manganese-enhanced *T*_1_ mapping as shown by the sensitivity of our data (Figure 3). The small intestinal wall of AMI model had lower Δ*R*_1_ than that of the sham-operated group (Figure 4).

Currently, the most accurate method for diagnosing AMI is based on mesenteric angiography. The gold standard for the diagnosis of AMI has been conventional catheter angiography that has higher sensitivity and accuracy [3]. However, it is an invasive, time-consuming, and technically complex procedure. Moreover, this diagnostic technique is unavailable at most hospitals leading to a critical delay. MDCT and CTA are advantageous over conventional angiography in that they not only delineate vascular structures but also show bowel wall changes and may exclude other causes of acute abdomen. Today, because it is less invasive and time-consuming, CTA has replaced conventional angiography as the gold standard in diagnosing AMI with sensitivity and specificity of 96% and 94%, respectively [1, 4, 26]. The magnetic resonance angiography (MRA) is a developing technique. Because of being noninvasive, the lack of radiation, and allergic risk related to iodinated contrast agents, MRA is the second choice for children and patients with azotemia [27]. There is a report that shows that MRA yielded sensitivity and specificity of 95% and 100%, respectively, for diagnosis of severe stenosis or occlusion of the origins of the celiac axes and superior mesenteric artery (SMA) [7]. However, this technique is limited to identification of more distally located occlusions. Based on current evidence, blood marker and peritoneal fluid analysis of laboratory findings are yet delayed and inaccurate to be an early diagnostic aid [28].

The literature reported that AMI is caused by an arterial embolus or thrombosis within the SMA in 60% to 70% of cases, nonocclusive ischemia in 20% to 30% of cases, and mesenteric vein thrombosis in 5% to 10% of cases [29]. There are some inevitable limits because of the fact that this technique is based on the perfusion of blood flow. Our present method is only applicable to that situation due to the occlusion of mesenteric artery. While Mn^2+^ flows to suffering small intestine along blood flow in the situation of the occlusion of mesenteric vein, this technique cannot discern normal and ischemic small intestines. Furthermore, this technique is also inappropriate for nonocclusive ischemia.

## 5. Conclusions

In conclusion, our investigation is the first to demonstrate that MEMRI at a low dose of Mn^2+^ reveals the differences of relaxivity between normal and ischemic small intestines associated with the occlusion of SMA. We hope that this exploratory research will provide additional information on preclinical and transnational models to promote diagnostic development of AMI.

## Figures and Tables

**Table 1:** Mean *R*_1_ values before and after Mn^2+^ infusion and mean Δ*R*_1_ for control group, sham-operated group, and AMI model (1/s).

Group	*R*_1_ (1/s)		Δ*R*_1_ (1/s)^a^
	Before Mn^2+^	After Mn^2+^	
Control	0.49 ± 0.033	2.54 ± 0.22	2.05 ± 0.19 (*n* = 6)
Sham-operated	0.48 ± 0.028	2.23 ± 0.19	1.75 ± 0.20 (*n* = 6)
AMI	0.49 ± 0.037	1.44 ± 0.16^b,c^	0.95 ± 0.13 (*n* = 6)^b,c^

^a^Values are expressed as mean ± SEM.

^b^ *P* < 0.01 among all groups (analysis of one-way ANOVA).

^c^ *P* < 0.01 compared with control group (unpaired two-tailed *t*-test).
